# Pediatric-Onset Multiple Sclerosis and Primary Headache: Is There a Link?

**DOI:** 10.3390/children12080963

**Published:** 2025-07-22

**Authors:** Giuseppe Tiralongo, Gabriele Monte, Michela A. N. Ferilli, Fabiana Ursitti, Giorgia Sforza, Claudia Ruscitto, Giuseppe Mazzeo, Alessandro Borrelli, Massimiliano Valeriani, Laura Papetti

**Affiliations:** 1 Academy of Pediatrics, Tor Vergata University of Rome, 00133 Rome, Italy; giuseppetiralongo95@gmail.com; 2 Developmental Neurology Unit, Bambino Gesù Children’s Hospital, IRCCS, 00165 Rome, Italy; gabriele.monte@opbg.net (G.M.); michela.ferilli@opbg.net (M.A.N.F.); fabiana.ursitti@opbg.net (F.U.); giorgia.sforza@opbg.net (G.S.); claudia.ruscitto@opbg.net (C.R.); massimiliano.valeriani@opbg.net (M.V.); 3 Faculty of Medicine and Surgery, Sapienza University of Rome, 00185 Rome, Italy; giuseppemazzeo94@gmail.com; 4 Child Neurology and Psychiatry Unit, Department of Wellbeing of Mental and Neurological, Dental and Sensory Organ Health, Policlinico Tor Vergata Foundation Hospital, 00133 Rome, Italy; 5 System Medicine Department, Tor Vergata University of Rome, 00133 Rome, Italy

**Keywords:** pediatric multiple sclerosis, headaches, migraine, childhood, POMS

## Abstract

Background: Pediatric-onset multiple sclerosis (POMS) is a rare but often more aggressive form of multiple sclerosis, associated with early cognitive impairment and significant impact on quality of life. Multiple sclerosis and primary headaches, particularly migraine, are well established in adults, but data on pediatric populations remain limited. Methods: The purpose of this retrospective study was to examine 64 POMS patients, divided into groups with and without headaches, to determine potential correlations between headache presence, age at POMS onset, and MRI lesion burden. Results: Headaches were reported by 78% of patients, predominantly migraines (68%), with a significantly higher prevalence in females (74%). No significant differences were found in age at MS onset or lesion load on brain MRI between patients with and without headaches. Among those with headaches, migraines represented a higher frequency of attacks and a greater need for prophylactic treatment compared to other headache types. Headache characteristics, including pain location and associated symptoms, showed no correlation with age at MS onset or lesion burden. Conclusions: These findings indicate that while headaches are common in POMS and more frequent in females, their presence and features do not appear to directly influence the clinical or neuroradiological course of the disease. Further research with larger cohorts and longitudinal follow-up is warranted to better understand the underlying mechanisms and long-term impact of headaches in pediatric MS.

## 1. Introduction

Multiple sclerosis (MS) is a chronic inflammatory disease of the central nervous system (CNS), characterized by demyelinating lesions in the brain, spinal cord, and optic nerves. Pediatric-onset MS (POMS), diagnosed in individuals under the age of 18, is relatively uncommon compared to adult-onset MS and presents unique challenges in diagnosis, treatment, and long-term management. In children, the disease may follow a more aggressive course, with faster progression and more severe symptoms, often leading to early cognitive impairment and a significant impact on quality of life—particularly in areas such as social development and academic performance. Numerous studies have shown that comorbid conditions, including stroke, epilepsy, and headache, can further exacerbate the disease burden in MS patients [1,2].

A meta-analysis published in 2021 reported a pooled migraine prevalence of 30% among MS patients—more than twice the rate observed in the general population [3]. The potential causal relationship between migraine and MS has been debated for decades. Some researchers suggest that migraine may be a risk factor for the development of MS [4,5], while others propose that MS may either co-occur with or trigger migraine [6]. The two conditions share several risk factors, including female sex, European ancestry, smoking, obesity, vitamin D deficiency [7,8], and adverse childhood experiences [9,10]. Recent evidence also points to a certain degree of genetic overlap [11].

From a pathophysiological perspective, the mechanisms underlying MS may also contribute to migraine symptoms. Both conditions involve neuroinflammatory processes, and demyelinating lesions—particularly those affecting regions such as the brainstem or periaqueductal gray matter—have been associated with increased headache activity [12]. Moreover, disease-modifying therapies used in MS, including interferon beta and fingolimod, have been linked to an increased risk of headache as a side effect. Additional factors common in MS, such as fatigue, stress, and depression, may also contribute to the higher prevalence of migraine [13,14].

Although limited studies have examined headaches in POMS, to date no large-scale analysis has evaluated correlations with MRI lesion burden or disease onset in this population. To address this gap, we conducted a retrospective study to assess the impact of primary headaches on the clinical, neuroradiological, and therapeutic course of patients with POMS. Specifically, in this study we investigated whether the presence of headache in the clinical history could be associated with a younger age at onset of POMS and a higher number of lesions on brain MRI. Furthermore, we examined the characteristics of headache in POMS patients who reported this symptom, aiming to understand whether these features were related to age at onset and number of brain MRI lesions.

## 2. Materials and Methods

We included in our study all patients followed at our center who had received a diagnosis of pediatric-onset multiple sclerosis (POMS). All patients were contacted by telephone to complete a questionnaire investigating their history of headaches, which may have introduced recall bias and affected diagnostic accuracy. This limitation should be considered when interpreting the results. Patients who reported experiencing headaches were invited to a screening visit to further evaluate the characteristics of their headaches. Data related to POMS were collected retrospectively from our center’s databases, while headache-related data were collected during these visits between January and April 2024.

The diagnosis of POMS was based on the McDonald criteria valid at the time of clinical onset (2005–2017) [15]. These criteria combine clinical signs with MRI evidence of dissemination in space and time of CNS lesions. For patients under 18 years of age, differential diagnosis ruled out other demyelinating syndromes such as MOG antibody-associated disease, neuromyelitis optica spectrum disorder, and acute disseminated encephalomyelitis. Typical MS lesion patterns and cerebrospinal fluid (CSF) analysis, including the presence of oligoclonal bands, were also considered. Clinical data, CSF and blood biochemical tests, and neuroradiological and neurophysiological data were available for all patients and were consistent with a diagnosis of POMS.

The definition and classification of primary headaches were based on the diagnostic criteria outlined in the International Classification of Headache Disorders, Third Edition (ICHD-3) [16]. According to the ICHD-3, primary headaches are not attributed to another medical condition and include migraine (with or without aura), tension-type headache, and trigeminal autonomic cephalalgias (TACs). Each diagnosis was made based on detailed clinical criteria such as attack frequency, pain characteristics (e.g., quality, intensity, and location), associated symptoms (e.g., photophobia, phonophobia, nausea), and duration of episodes. Secondary causes of headache were systematically excluded through clinical assessment and diagnostic workup, ensuring that only headaches fulfilling ICHD-3 criteria for primary forms were included in the analysis.

We divided our population into two groups: the first consisted of POMS patients with headache, and the second of POMS patients without headache.

The POMS-related data collected included demographic variables (sex, age), clinical variables (age at onset of MS and age at the screening visit), neuroradiological variables (lesion count), and disease-modifying therapy (DMT). For the analysis of MRI data, the neuroimaging exam closest to the onset of headache was considered. For patients with a history of headache preceding that of POMS, the first MRI performed at the time of clinical onset of MS was considered.

Lesion count was categorized as low when fewer than 10 typical MS lesions were present and high when more than 10 were observed. The threshold of >10 lesions was chosen arbitrarily due to the lack of standardized or validated cut-offs for POMS populations. Future studies should validate this definition using clinical correlates.

On MRI, typical MS lesions appear as hyperintense, round or oval demyelinating lesions on T2-weighted images, and as hypointense on T1-weighted images. Lesions may be found throughout the CNS, including the brain (periventricular, subcortical, and cortical regions), spinal cord, and optic nerves [17].

For each patient with headache, we analyzed the following variables: headache diagnosis (migraine with or without aura, tension-type headache, or TACs); side and location of pain; pain quality (throbbing, stabbing, or constrictive); presence of associated symptoms (photophobia, phonophobia, nausea, vomiting); and monthly headache days (MHDs). Based on MHDs, patients with headache were classified as follows: low-frequency episodic (LFE) headache (<8 MHDs), high-frequency episodic (HFE) headache (8–14 MHDs), and chronic headache (≥15 MHDs). We also evaluated headache treatment-related variables, including response to analgesics and prophylactic therapy.

Clinical and demographic measures were reported as absolute values and frequencies (%), means and standard deviations (SDs) for normally distributed data, or medians and interquartile ranges (IQRs) for non-normally distributed data.

The chi-square test was used to analyze categorical variables, while ANOVA was used for continuous variables.

Univariate analysis was conducted to examine the relationship between the presence or absence of headache and MRI lesion count as well as age at POMS onset.

The primary hypothesis we aimed to test was that the presence of headache might be a negative prognostic factor, associated with a higher MRI lesion count and a younger age of onset of POMS.

Multivariate analysis was used to assess, among POMS patients with headache, the possible associations between sex, headache diagnosis, headache features (attack frequency, photophobia, phonophobia, nausea/vomiting), and MRI lesion count.

Multivariate analysis was performed using a generalized linear model (GLM) with a cumulative link and proportional odds assumption. This model was chosen to accommodate the ordinal nature of lesion burden and attack frequency. However, formal testing of model assumptions—including multicollinearity and proportionality—was not performed, which should be considered a limitation of the study.

Statistical significance was determined using p-values, with values < 0.05 considered significant. Bonferroni correction was applied to adjust for multiple comparisons.

No formal power analysis was conducted, which may limit the ability to detect small but clinically meaningful differences. This limitation should be considered when interpreting negative findings.

## 3. Results

### 3.1. All POMS Patients

The number of patients in our POMS population was 64. The ratio of females to males was 2:1, with 43 females and 21 males. The mean age for patients with headache who attended the screening visit was 17.77 years old (with an average age of 8.71 and a maximum of 25.39), with an SDS of 3.38. The age at onset of POMS was 13.6 years (minimum 5.4, maximum 17.8; SDS 2.6). At the time of recruitment, patients had a mean duration of POMS of approximately 4.2 years ±2 standard deviations. Our POMS population’s demographics are similar to those reported in the literature, making it suitable to be considered an ideal POMS population [18]. On MRI, 23 patients (35.9%) showed a low count of lesions, while 41 patients (64%) had a high count of lesions. Natalizumab (28%), fingolimod (23%), and ocrelizumab (22%) were the most frequently used therapies, while cladribine (1.6%), interferon beta (5%), and dimethyl fumarate (5%) were the least used. More details are shown in Table 1.

### 3.2. POMS Patients with Headaches

**Figure 1:**
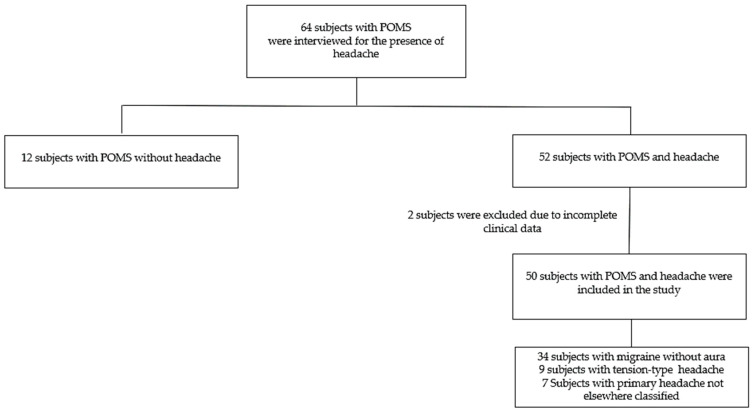
Flowchart of recruitment of subjects.

Fifty patients (78%) reported suffering from headaches and were included in the study (Figure 1). The mean age at headache onset was 14.6 years (range: 7.3–21.5 years; SD ± 2.3). In 85% of the subjects analyzed, the onset of headache occurred after the diagnosis of POMS, on average about one year after the diagnosis. For patients with a prior history of headache, the qualitative characteristics remained the same as before the diagnosis of POMS.

POMS patients with headache were more likely to have a parental history of headache compared to those without headache (*p* < 0.05). Regarding pain laterality, 68% of patients reported bilateral pain, while 32% reported unilateral pain, with 10% describing the pain as consistently localized to one side. The most frequently reported location of pain was the temporal region (74%).

The majority of patients (62%) described the pain as throbbing, followed by constrictive pain (34%), while only a small proportion (4%) reported stabbing pain.

Approximately half of the patients with headaches reported photophobia (56%) and phonophobia (44%), while 32% experienced nausea or vomiting. According to the ICHD-3 classification, 68% of POMS patients with headache were diagnosed with migraine, 18% with tension-type headache, and 14% with a primary headache not otherwise classified. The mean duration of headache attacks was 519 ± 80 min (approximately 8 h). No significant differences were observed between high-frequency episodic (HFE; 32%) and low-frequency episodic (LFE; 54%) headaches; 14% of patients met the criteria for chronic headache.

In terms of treatment, non-steroidal anti-inflammatory drugs (NSAIDs) were the most commonly used medications for managing acute headache attacks, followed by acetaminophen. Only three patients were treated with amitriptyline as a prophylactic therapy. All reported improvement, with a 50% reduction in the number of monthly headache attacks after three months of treatment. Additional details are provided in Table 2.

**Figure 2:**
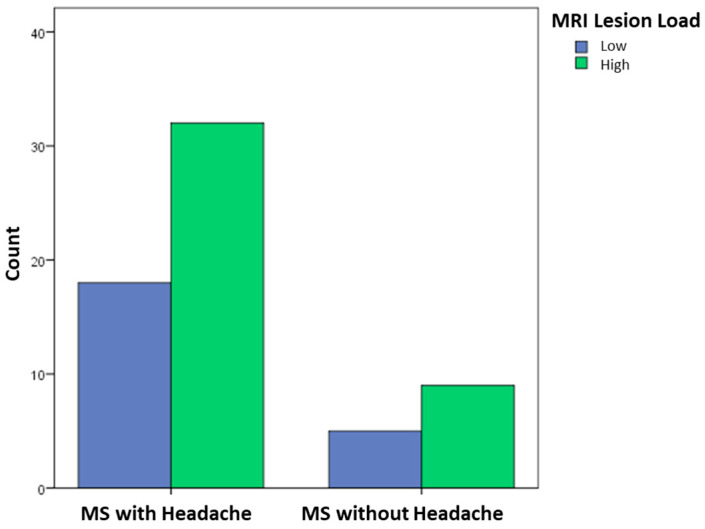
Distribution of lesion load on MRI in MS patients with and without headache.

No significant differences were found between POMS patients with and without headache. Specifically, there was no statistically significant difference in the age at POMS onset between those with headache (13.7 years) and those without (13.4 years). Similarly, there was no significant association between MRI lesion count and the presence of headache: a high lesion count was observed in 64% of patients with headache and in 64.3% of those without (*p* > 0.05) (Figure 2).

Female POMS patients with headache were significantly more common than male patients (females: 37/50, 74%; males: 13/50, 26%; *p* < 0.05).

We categorized patients based on their headache diagnosis (migraine, tension-type headache, and headache not otherwise classified) and found no significant differences in MRI lesion count across these groups. However, patients with migraine experienced a higher frequency of attacks compared to those with tension-type headache (*p* < 0.05) and were more likely to require prophylactic therapy (*p* < 0.05).

No significant correlations were found between age at POMS onset or MRI lesion count and headache characteristics, including headache type, side and location of pain, pain quality, associated symptoms, frequency of attacks, or response to analgesics and prophylactic treatment.

The results of the multivariate analysis evaluating the relationship between MRI lesion count and headache characteristics are summarized in Table 3. The only variable significantly associated with lesion count was female sex.

## 4. Discussion

Primary headaches and multiple sclerosis (MS) are two distinct yet disabling neurological disorders that significantly affect both patients and caregivers, resulting in considerable disability and reduced quality of life. In recent decades, there has been growing interest in exploring potential links between these conditions, driven by various clinical and epidemiological observations.

Primary headaches, such as migraine and tension-type headache, are characterized by recurrent episodes of head pain without an identifiable underlying condition. MS, in contrast, is a leading cause of neurological disability in young adults, with a pathogenesis shaped by complex genetic, environmental, and immunological interactions.

Pediatric-onset MS (POMS) is relatively rare compared to adult-onset MS and differs not only in the early age of onset but also in terms of disease severity, lesion burden, early cognitive impairment, and a higher relapse frequency in the initial phases. The epidemiological and clinical impact of headaches—particularly migraine without aura—on adult-onset MS is well documented, with a large proportion of adult MS patients reporting headache. Two large meta-analyses estimated that approximately 30% of MS patients suffer from migraine [3,19], with study populations predominantly comprising Caucasian females diagnosed in early adulthood. Kister et al. (2012) reported a 29% increased risk of MS among females with pre-existing migraine [5].

Based on such findings, some researchers have proposed that migraine may act as a risk factor for MS. However, this association remains debated and may be partly attributable to diagnostic bias, given the shared epidemiological profiles of both disorders (e.g., young, white females) and the tendency of migraine sufferers to undergo brain imaging, which could detect non-specific white matter changes mistakenly attributed to MS. An alternative hypothesis is that migraine-like headaches may be an early symptom or part of an MS prodromal phase [20]. Nevertheless, the prevailing view is that, since migraines usually precede MS onset by several years and are uncommon at disease presentation, they are more likely a pre-existing comorbidity rather than a prodromal manifestation [21].

Despite the extensive literature on primary headaches and MS in adults, data on pediatric patients remain limited [3,4,5,6]. Mariotti et al. described a case of a very young girl in whom severe headache was both the presenting symptom and a recurring feature during relapses, although accompanied by other neurological deficits [22]. A brainstem lesion involving pain-processing areas, accompanied by swelling and possible meningeal irritation in the periaqueductal gray, was suggested as the underlying mechanism [23].

Radiologically isolated syndrome (RIS) has also been increasingly reported in pediatric populations, particularly in children undergoing imaging for headaches. In a longitudinal multicenter study, Makhani et al. (2017) found that 42% of children with RIS developed a first clinical event, and 61% showed radiological progression—both higher than rates observed in adults [24]. Although our study focused exclusively on children with a confirmed MS diagnosis, these findings underscore the importance of considering RIS when evaluating pediatric patients presenting with headache. Nevertheless, the exact role of headache in RIS—either as a presenting symptom or as a predictor of disease progression—remains unclear. Further prospective studies are warranted to determine which children with RIS are at greatest risk of conversion and whether early treatment may be beneficial.

Given these considerations, we evaluated our POMS cohort to assess the potential impact of headache, its characteristics, and its treatments on disease onset and progression.

Our data did not support a significant association between headache presence and disease burden, although the limited sample size and retrospective design preclude definitive conclusions. Headache occurrence may be influenced by unmeasured variables outside of MS activity or lesion burden, such as hormonal or psychological factors, which were not assessed in this study.

However, among POMS patients with headache, a significant sex difference was observed. Female patients were more likely to experience headache than males (74% vs. 26%, *p* < 0.05), a pattern consistent with epidemiological trends in the general population [25,26]. This supports the hypothesis that sex-related biological mechanisms, including hormonal influences, may contribute to increased headache susceptibility in both POMS and non-MS populations [27,28,29,30].

Further classification of headache types (migraine, tension-type headache, and headache not otherwise specified) revealed no significant differences in distribution based on disease-modifying therapy (DMT). However, patients with migraine reported a higher attack frequency compared to those with tension-type headache (*p* < 0.05) and were more likely to require prophylactic treatment (*p* < 0.05), suggesting a greater headache burden in this subgroup.

No significant associations were found between age at POMS onset and various headache characteristics, including location, side, pain quality, accompanying symptoms, attack frequency, or treatment response. Similarly, no relationships were observed between lesion burden, age, or type of DMT and any of the headache-related variables.

Unlike other authors [31], in our cohort we did not find differences in the number of gadolinium-enhancing lesions between POMS patients with headache and those without headache.

This suggests that headache occurrence may be influenced by unmeasured factors unrelated to MS activity or lesion load, such as hormonal or psychological variables, which were not assessed in our study.

Regarding treatment, most patients in our cohort were receiving off-label DMTs for pediatric MS during the study period. Fingolimod, which was approved for pediatric use, was administered in 23% of cases. Although teriflunomide has been approved by the EMA for pediatric MS, none of the patients in our cohort received it, possibly due to local prescribing preferences and the lack of FDA approval. The variability in DMT use may have influenced headache occurrence, either as a side effect or as a comorbid condition, representing another limitation in interpreting our findings.

The retrospective nature of our study, the initial selection of patients using a telephone questionnaire, and the low number of recruited subjects are all limitations that must be considered. A power analysis was not conducted, which may limit the ability to detect small but clinically meaningful differences. Furthermore, the occurrence of headache may be influenced by unmeasured variables outside of MS activity or lesion burden, such as hormonal or psychological factors, which were not assessed in this study.

Overall, our results emphasize the complexity of the relationship between headache and POMS. The strong female predominance aligns with known epidemiological patterns, while the absence of associations with lesion burden or DMT suggests that other factors—possibly genetic, hormonal, or environmental—may underlie headache pathophysiology in POMS [32]. Future studies with larger, prospective, multicenter cohorts are needed to further explore these relationships and identify potential mechanisms.

## 5. Conclusions

Our data indicate that primary headache does not appear to be related to key features of POMS, such as age at onset, type of treatment, or medication use. Thus, the progression of the two conditions appears to be independent. To obtain more robust conclusions, future research should involve multicenter, prospective studies with larger sample sizes and longer follow-up periods.

Further investigation is also warranted to assess the impact of primary headache on neuropsychological outcomes (e.g., cognitive, adaptive, and executive functions), school attendance, and overall quality of life for both patients and caregivers. In addition, studies should explore changes in cerebrospinal fluid and systemic inflammatory markers.

Although a direct causal link between primary headaches and MS has not been confirmed, their co-occurrence suggests that MS patients should be monitored for migraine symptoms. Appropriate management of both conditions may improve quality of life and functional outcomes.

## Figures and Tables

**Table 1:** Demographic and clinical characteristics of POMS patients with and without headaches.

Variable	POMS with Headache	POMS Without Headache	Sig.
Sex (female, male %)	74%, 26%	42.9%, 26%	*
Age at onset of POMS (mean ± SDS)	13.7 ± 2.6	13.4 ± 2.6	–
Lesion count on MRI (low, high)	36%, 64%	35.7%, 64.3%	–
Gadolinium-enhancing lesions (mean ± SDS)	0.9 ± 0.6	0.7 ± 0.3	–
Familiarity with headaches	72%	42.9%	*
DMT (%)			–
INFb sc	0%	7.1%	
INFb im	2%	7.1%	
DMF	4%	7.1%	
NTZ	24%	42.9%	
RTX	16%	14.3%	
FNG	28%	7.1%	
OCZ	24%	14.3%	
CLD	2%	1.6%	

CLD: cladribine; DMF: dimethyl fumarate; DMT: disease-modifying therapy; FNG: fingolimod; INFb: interferon beta; im: intramuscular; NTZ: Natalizumab; OCZ: ocrelizumab; RTX: rituximab; sc: subcutaneous; SDS: standard deviation score; Sig: significance; *: *p* < 0.05; –: *p* > 0.05.

**Table 2:** Clinical profile and treatment approaches for headaches in POMS.

Feature of Headache	Frequency and Percentage (Total 50 Subjects)
Female/Male	37, 74%: 13, 26%
Mean age at onset of headache	14.6 years, SD ± 2.3
Diagnosis	Migraine without aura: 34, 68% Tension-type headache: 9, 18% Primary headache not elsewhere classified: 7, 14%
Frequency of MHDs	LFE: 27, 54% HFE: 16, 32% Chronic: 7, 14%
Quality of pain	Throbbing: 31, 62% Constrictive: 17, 34% Stabbing: 2, 4%
Pain side	Unilateral with side preference: 5, 10% Unilateral with no side preference: 11, 22% Bilateral: 34, 68%
Location of pain	Frontal: 9, 18% Temporal: 37, 74% Widespread: 4, 8%
Associated symptoms	Photophobia: 28, 56% Phonophobia: 22, 44% Nausea or vomiting: 12, 32%
Drugs for treating the attacks	Acetaminophen: 14, 28% Ibuprofen: 14, 38% Ketoprofen: 9, 18% Nimesulide: 1, 2% Ketoralac: 1, 2%
Prophylactic therapy for chronic subjects	Amitriptyline: 3, 100%

HFE: high-frequency episodic (8–14 MHDs), and chronic (≥15 MHDs); LFE: low-frequency episodic (<8 MHDs); MHDs: monthly headache days.

**Table 3:** Results of the multivariate analysis performed to verify a relationship between MRI lesion count and headache characteristics.

Variable	Beta Coefficient	Standard Deviation	T	Sig.
Sex	1.3	0.6	3.9	0.04
Type of headache (migraine vs. TTH)	−0.217	0.14	−1.4	0.14
Frequency of headache attacks per month (high, low)	0.14	0.1	0.21	1.36
Photophobia	−0.72	0.17	−0.4	0.68
Phonophobia	0.37	0.18	1.9	0.06
Nausea or vomiting	0.16	0.16	0.96	0.34

## Data Availability

The data presented in this study are available on request from the corresponding author due to ethical restrictions related to patient confidentiality and the protection of sensitive personal health information.

## Abbreviations

The following abbreviations are used in this manuscript:

CLD	Cladribine
CNS	Central nervous system
DMF	Dimethyl fumarate
DMT	Disease-modifying therapy
FNG	Fingolimod
GLM	Generalized linear model
ICHD-3	The International Classification of Headache Disorders, 3rd edition
IQR	Interquartile range
INFb-im	Intramuscular interferon beta
INFb-sc	Subcutaneous interferon beta
HFE	High-frequency episodic
LFE	Low-frequency episodic
MHDs	Monthly headache days
MRI	Magnetic Resonance Imaging
MS	Multiple sclerosis
NSAIDs	Non-steroidal anti-inflammatory drugs
NTZ	Natalizumab
OCZ	Ocrelizumab
POMS	Pediatric-onset MS
RTX	Rituximab
SDS	Standard deviation score
TACs	Trigeminal autonomic cephalalgias

## References

[1] Moisset X., Ouchchane L., Guy N., Bayle D.J., Dallel R., Clavelou P. (2013). Migraine headaches and pain with neuropathic characteristics: Comorbid conditions in patients with multiple sclerosis. Pain.

[2] Marrie R.A., Fisk J.D., Fitzgerald K., Kowalec K., Maxwell C., Rotstein D., Salter A., Tremlett H. (2023). Etiology, effects and management of comorbidities in multiple sclerosis: Recent advances. Front. Immunol..

[3] Wang L., Zhang J., Deng Z.R., Zu M.D., Wang Y. (2021). The epidemiology of primary headaches in patients with multiple sclerosis. Brain Behav..

[4] Yusuf F.L.A., Ng B.C., Wijnands J.M.A., Kingwell E., Marrie R.A., Tremlett H. (2020). A systematic review of morbidities suggestive of the multiple sclerosis prodrome. Expert Rev. Neurother..

[5] Kister I., Munger K.L., Herbert J., Ascherio A. (2012). Increased risk of multiple sclerosis among women with migraine in the Nurses’ Health Study II. Mult. Scler..

[6] Schoeps V.A., Smith J.B., Langer-Gould A. (2025). Migraines, the multiple sclerosis prodrome, and multiple sclerosis susceptibility. Mult. Scler..

[7] Belbasis L., Bellou V., Evangelou E., Tzoulaki I. (2020). Environmental factors and risk of multiple sclerosis: Findings from meta-analyses and Mendelian randomization studies. Mult. Scler..

[8] Yuan S., Daghlas I., Larsson S.C. (2022). Alcohol, coffee consumption, and smoking in relation to migraine: A bidirectional Mendelian randomization study. Pain.

[9] Mansuri F., Nash M.C., Bakour C., Kip K. (2020). Adverse Childhood Experiences (ACEs) and Headaches Among Children: A Cross-Sectional Analysis. Headache.

[10] Eid K., Torkildsen Ø., Aarseth J., Aalstad M., Bhan A., Celius E.G., Cortese M., Daltveit A.K., Holmøy T., Myhr K.M. (2022). Association of adverse childhood experiences with the development of multiple sclerosis. J. Neurol. Neurosurg. Psychiatry.

[11] Horton M.K., Robinson S.C., Shao X., Quach H., Quach D., Choudhary V., Bellesis K.H., Dorin P., Mei J., Chinn T. (2023). Cross-Trait Mendelian Randomization Study to Investigate Whether Migraine Is a Risk Factor for Multiple Sclerosis. Neurology.

[12] Gee J.R., Chang J., Dublin A.B., Vijayan N. (2005). The association of brainstem lesions with migraine-like headache: An imaging study of multiple sclerosis. Headache.

[13] Gebhardt M., Kropp P., Hoffmann F., Zettl U.K. (2019). Headache in the course of multiple sclerosis: A prospective study. J. Neural Transm..

[14] Husain F., Pardo G., Rabadi M. (2018). Headache and Its Management in Patients with Multiple Sclerosis. Curr. Treat. Options Neurol..

[15] Thompson A.J., Banwell B.L., Barkhof F., Carroll W.M., Coetzee T., Comi G., Correale J., Fazekas F., Filippi M., Freedman M.S. (2018). Diagnosis of multiple sclerosis: 2017 revisions of the McDonald criteria. Lancet Neurol..

[16] International Headache Society (2018). The International Classification of Headache Disorders, 3rd edition (ICHD-3). Cephalalgia.

[17] Filippi M., Preziosa P., Banwell B.L., Barkhof F., Ciccarelli O., De Stefano N., Geurts J.J.G., Paul F., Reich D.S., Toosy A.T. (2019). Assessment of lesions on magnetic resonance imaging in multiple sclerosis: Practical guidelines. Brain A J. Neurol..

[18] Nourbakhsh B., Cordano C., Asteggiano C., Ruprecht K., Otto C., Rutatangwa A., Lui A., Hart J., Flanagan E.P., James J.A. (2021). Multiple Sclerosis Is Rare in Epstein-Barr Virus-Seronegative Children with Central Nervous System Inflammatory Demyelination. Ann. Neurol..

[19] Mirmosayyeb O., Barzegar M., Nehzat N., Shaygannejad V., Sahraian M.A., Ghajarzadeh M. (2020). The prevalence of migraine in multiple sclerosis (MS): A systematic review and meta-analysis. J. Clin. Neurosci..

[20] Tremlett H., Munger K.L., Makhani N. (2022). The Multiple Sclerosis Prodrome: Evidence to Action. Front. Neurol..

[21] Gklinos P., Mitsikostas D.D. (2024). Headache disorders in multiple sclerosis: Is there an association? A systematic review and meta-analysis. Mult. Scler. Relat. Disord..

[22] Mariotti P., Nociti V., Cianfoni A., Stefanini C., De Rose P., Martinelli D., Dittoni S., Vollono C., Batocchi A.P., Della Marca G. (2010). Migraine-like headache and status migrainosus as attacks of multiple sclerosis in a child. Pediatrics.

[23] Tortorella P., Rocca M.A., Colombo B., Annovazzi P., Comi G., Filippi M. (2006). Assessment of MRI abnormalities of the brainstem from patients with migraine and multiple sclerosis. J. Neurol. Sci..

[24] Makhani N., Lebrun C., Siva A., Brassat D., Carra Dallière C., de Seze J., Du W., Durand Dubief F., Kantarci O., Langille M. (2017). Radiologically isolated syndrome in children: Clinical and radilogic outcomes. Neurol. Neuroimmunol. Neuroinflamm..

[25] Ursitti F., Valeriani M. (2023). Migraine in childhood: Gender differences. Eur. J. Paediatr. Neurol..

[26] Wilcox S.L., Ludwick A.M., Lebel A., Borsook D. (2018). Age- and sex-related differences in the presentation of paediatric migraine: A retrospective cohort study. Cephalalgia Int. J. Headache.

[27] Krause D.N., Warfvinge K., Haanes K.A., Edvinsson L. (2021). Hormonal influences in migraine—Interactions of oestrogen, oxytocin and CGRP. Nat. Rev. Neurol..

[28] Singh S., Kopruszinski C.M., Watanabe M., Dodick D.W., Navratilova E., Porreca F. (2024). Female-selective mechanisms promoting migraine. J. Headache Pain.

[29] Langille M.M., Rutatangwa A., Francisco C. (2019). Pediatric Multiple Sclerosis: A Review. Adv. Pediatr..

[30] Murgia F., Giagnoni F., Lorefice L., Caria P., Dettori T., D’Alterio M.N., Angioni S., Hendren A.J., Caboni P., Pibiri M. (2022). Sex Hormones as Key Modulators of the Immune Response in Multiple Sclerosis: A Review. Biomedicines.

[31] Graziano E., Hagemeier J., Weinstock-Guttman B., Ramasamy D.P., Zivadinov R. (2015). Increased contrast enhancing lesion activity in relapsing-remitting multiple sclerosis migraine patients. Neuroimage Clin..

[32] Cappa R., Theroux L., Brenton J.N. (2017). Pediatric Multiple Sclerosis: Genes, Environment, and a Comprehensive Therapeutic Approach. Pediatr. Neurol..

